# Sensitizing Colorectal Cancer to PARP Inhibitors: Biomarkers, Mechanisms, and Combination Strategies

**DOI:** 10.3390/ijms27188127

**Published:** 2026-09-12

**Authors:** Mariam Elesnawy, Eman Masood, Adviti Naik

**Affiliations:** Biological Sciences, Carnegie Mellon University Qatar, Doha 24866, Qatar

**Keywords:** PARP inhibitors, poly-ADP ribose polymerase, colorectal cancer, DNA damage repair, combination therapy, personalized therapy

## Abstract

Poly(ADP-ribose) polymerase inhibitors (PARPis) have transformed the treatment landscape of homologous recombination-deficient malignancies, particularly *BRCA1/2*-mutant breast, ovarian, pancreatic, and prostate cancers. In contrast, clinical studies evaluating PARPis in broadly selected colorectal cancer (CRC) patients have yielded disappointing outcomes despite evidence that a subset of CRCs harbor DNA damage response (DDR) defects and homologous recombination deficiency (HRD)-associated mutational signatures. These observations highlight the need for improved patient stratification and the development of strategies to enhance PARPi sensitivity in CRC. In this review, we integrate CRC-specific evidence with mechanistic and preclinical findings from other malignancies and discuss candidate biomarkers associated with PARPi responsiveness, including *TP53*, RAD51, and *MRE11*, and examine their potential utility in identifying CRC patient populations most likely to benefit from PARP inhibition. We further summarize therapeutic approaches aimed at inducing “BRCAness” and sensitizing CRC cells to PARPis through epigenetic modulation, targeting cell-cycle checkpoint regulators, inhibiting growth factor signaling pathways, and exploiting metabolic vulnerabilities. Emerging strategies involving polyamine inhibition and modulation of NAD+ metabolism have been specifically highlighted for their potential role in therapeutic response. Collectively, these approaches provide a framework for investigating mechanistically compelling opportunities to overcome intrinsic and acquired resistance to PARPis and expand the clinical utility of DDR-targeted therapies in CRC. A deeper mechanistic understanding of PARPi response and resistance, in addition to extensive investigations in CRC-specific models and molecularly selected clinical cohorts, will facilitate the development of biomarker-driven combination therapies and improve outcomes for patients with CRC.

## 1. Introduction

Poly(ADP-ribose) polymerases (PARPs) are a family of proteins that are essential for cellular responses to DNA damage. The human PARP family comprises 18 known isoenzymes, with PARP1 and PARP2 being the most studied and ubiquitously expressed. PARP1/2 utilize Nicotinamide Adenine Dinucleotide (NAD+) as a substrate to generate poly(ADP-ribose) (PAR) polymers, and they have distinct functions in DNA repair, transcription regulation, and metabolic processes [1]. Despite their similarities, PARP1 and PARP2 exhibit functional differences as they target distinct proteins, suggesting they serve unique biological roles [2].

In this review, we focus on PARP1, an enzyme that identifies and binds to single and double strand DNA breaks through its DNA-binding domain. Following the detection of DNA damage, it becomes activated and catalyzes the formation of PAR chains on itself and other acceptor proteins. PAR signaling subsequently facilitates the recruitment of chromatin-remodeling proteins to sites of DNA damage through distinct mechanisms. ALC1/CHD1L is recruited through direct binding to PAR, whereas CHD4 recruitment is PAR-dependent but occurs indirectly through enhanced binding to PAR-induced relaxed chromatin [3,4,5]. In addition, PAR synthesized following DNA damage promotes the recruitment of the MRN complex to sites of DNA damage, which consists of MRE11 Homolog, Double-Strand Break Repair Nuclease (MRE11), RAD50 Double-Strand Break Repair Protein (RAD50), and Nibrin (NBS1). This complex recognizes DNA double-strand breaks (DSBs) [6,7,8].

**Figure 1:**
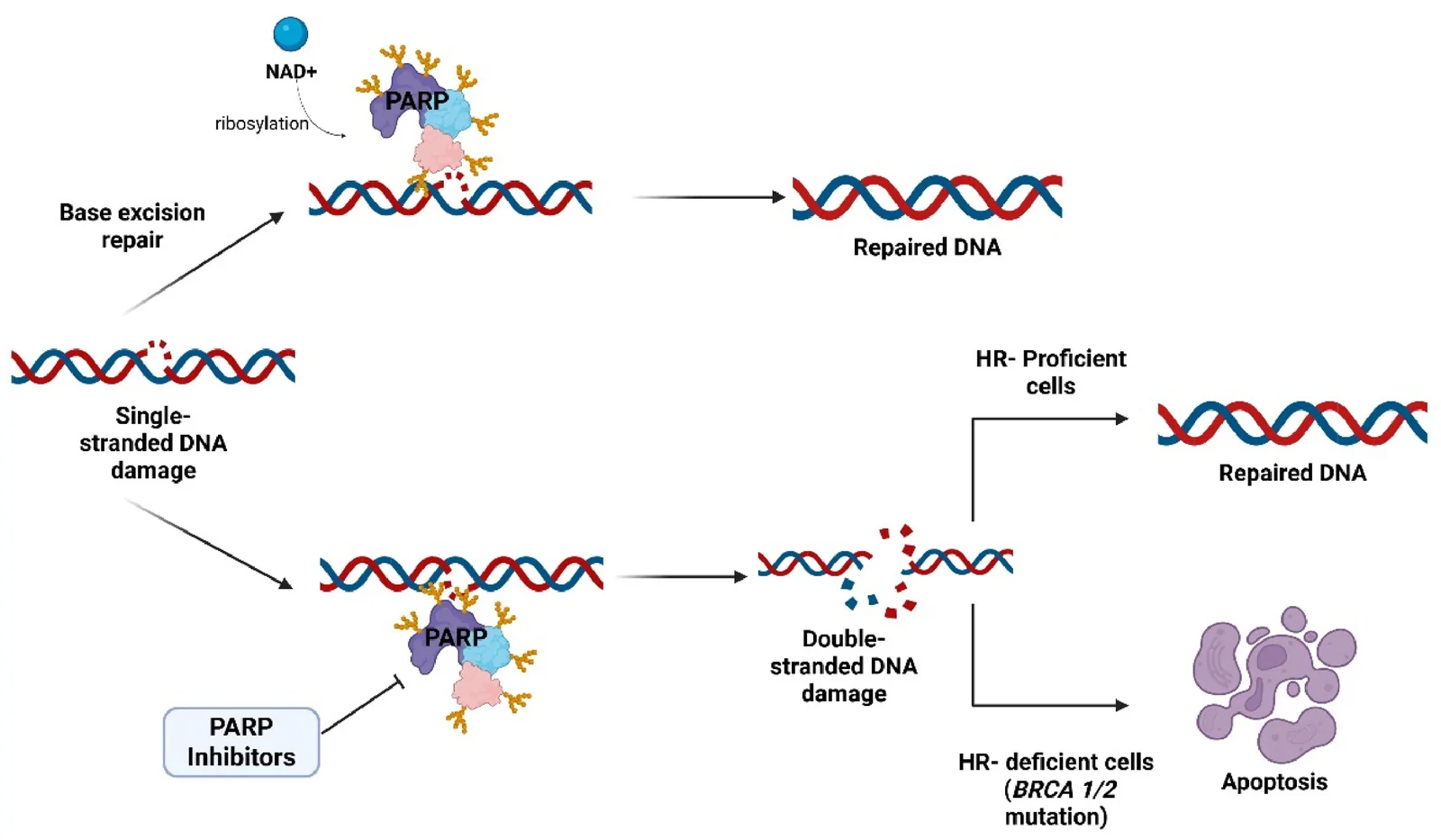
**Mechanism of PARPi action in HR-proficient and HR-deficient tumors.** PARP inhibition prevents SSB repair, leading to replication fork collapse and formation of double-strand DNA breaks (DSBs). HR-proficient cells repair DSBs through homologous recombination and survive, whereas HR-deficient cells fail to repair the damage, resulting in PARPi-induced synthetic lethality and cell death. Blunt arrow (⊣) represents inhibition. Created in BioRender. Masood, E. (2026) Available online: https://BioRender.com/rukv8or (accessed on 30 June 2026).

Differential PARP1 expression influences tumor characteristics and responses to treatment. Elevated levels of PARP1 are frequently correlated with aggressive forms of breast, ovarian, and pancreatic cancers, as well as resistance to therapies, especially in the context of *BRCA* mutations, as will be discussed later. In contrast, reduced PARP1 and PARP2 protein expression has been reported in non-small-cell lung cancer (NSCLC) compared with non-tumor lung tissue, despite evidence that PARP activity may remain elevated in specific clinical contexts, such as in patients with underlying chronic obstructive pulmonary disease (COPD) [9]. The importance of PARP expression and function in cancer has led to PARP inhibitors (PARPis) emerging as a promising therapeutic strategy, and, as such, PARPis structurally mimic the nicotinamide portion of NAD+, which serves as the natural substrate for PARP. This allows PARPis to competitively bind to the catalytic sites of PARP1 and PARP2, inhibiting their enzymatic functions and resulting in an accumulation of DNA damage due to the failure of repair mechanisms [10] (Figure 1). Examples of PARPis include olaparib, niraparib, rucaparib, talazoparib, and fuzuloparib. However, certain PARPis, such as pamiparib, also extend beyond the nicotinamide binding pocket into the neighboring ADP-ribose region of the NAD+ binding site, providing a strategic advantage in terms of selectivity and potency [11].

Upon binding to the catalytic site, PARPis not only block enzyme activity, but also stabilize the formation of a ternary complex with the damaged DNA, effectively “trapping” PARP at the site of the lesion: an additional mechanism contrubuting to PARPi cytotoxicity (Figure 1). This PARP–DNA complex becomes sufficiently harmful, leading to cell death, particularly in cells lacking BRCA-mediated repair capabilities [12]. Different PARPis vary in their trapping effectiveness, which correlates with their antitumor activity (Table 1), thereby underscoring the significance of both catalytic inhibition and PARP trapping in the design of efficient PARPis [13].

As this is a narrative review, the relevant literature was identified through searches of PubMed and Google Scholar, with ClinicalTrials.gov additionally searched for prospective clinical studies. The literature and trial evidence was updated through August 2026, and reference lists of relevant primary studies and reviews were additionally examined.

## 2. Synthetic Lethality

Landmark research conducted by Farmer et al. [35] and Bryant et al. [36] in 2005 established the principle of synthetic lethality: the mechanism by which PARPis function. They demonstrated that inhibiting PARP function can specifically kill cancer cells deficient in *BRCA1/2* key genes involved in DNA damage repair and genomic stability, due to their reliance on the PARP pathway for repair of DNA damage and, hence, survival.

In *BRCA1* or *BRCA2*-mutant cancers, such as breast and ovarian cancers, the pathway for homologous recombination (HR) is defective, and therefore, synthetic lethality by PARPis mainly relies on hampered base excision repair (BER), causing DNA damage and death. BERs damaged bases and single-strand DNA lesions. Failure of BER can indirectly promote replication-associated DSB formation and exacerbate genomic instability. The interplay of HR, BER, and non-homologous end joining (NHEJ) explains the basis for PARPi-induced synthetic lethality in cancer therapies. Although NHEJ functions throughout the cell cycle, it is the predominant double-strand break repair pathway during the G1 phase, when a sister chromatid is unavailable for homologous recombination. Compared with HR, NHEJ is inherently more error-prone and can introduce mutations during DNA repair [37,38]. Cancer cells often rely on NHEJ when HR is compromised, which can perpetuate genetic instability as HR provides a high-fidelity repair mechanism predominantly used during the S and G2 phases of the cell cycle. In contrast, this synergy between the DNA repair pathways allows cancer cells to adaptively shift among repair mechanisms in response to genotoxic stresses, which allows for targeting these vulnerabilities [39]. Therefore, PARPis function by targeting DNA repair pathways that *BRCA1/2*-mutant tumors are dependent upon (Figure 1). Inhibition of key components of these pathways leads to excessive DNA damage, triggering subsequent critical levels of genomic instability, mitotic catastrophe and ultimately cell death [40,41].

Although PARPi-mediated synthetic lethality has traditionally been explained by the conversion of unrepaired single-strand lesions into double-strand breaks following replication-fork collapse, emerging evidence indicates that this model is not sufficient to explain all PARPi-induced cytotoxicity. Accumulation of post-replicative single-stranded DNA (ssDNA) gaps has emerged as an important determinant of PARPi sensitivity, particularly in BRCA-deficient cells, while suppression of replication gaps can promote PARPi resistance [42]. In addition, PARP1 functions with the replisome factors TIMELESS and TIPIN to protect replication forks from transcription–replication conflicts, and PARP inhibition can promote DNA damage arising from unresolved transcription–replication conflicts in HR-deficient cells [43].

In contrast, BRCAness is a feature of certain tumor types that do not carry mutations in the *BRCA1* or *BRCA2* genes, but exhibit similar DNA repair deficiencies, possibly making such tumors susceptible to PARP inhibition. These tumors may exhibit phenotypic or molecular features resembling BRCA1/2-deficient tumors, collectively described as ‘BRCAness’ [44,45].

## 3. Success of PARPis in Breast, Ovarian and Prostate Cancers

PARPis have demonstrated clinical efficacy in *BRCA1/2*-mutation cancers, as mentioned above. This is most notable in breast and ovarian cancers. Breast cancer is the second most common cancer worldwide and the first leading cause of cancer-related deaths in women globally, whereas ovarian cancer accounts for 3.4% of cancer cases in women [46].

Since PARPis have emerged, they have been used as single agents, in combination with cytotoxic therapy, or in combination with ionizing radiation, mainly in *BRCA1/2*-mutated or HRD-positive cancers. Olaparib was the first PARPi to show activity in BRCA-related ovarian and breast cancers. Prior to its FDA approval, olaparib had demonstrated efficacy as a single agent as well as in combination with DNA damaging and anti-angiogenesis agents. In 2017, a phase III study (OlympiAD, (NCT02000622)) in locally advanced or metastatic HER2-negative, *BRCA1/2*-mutation-associated breast cancer patients showed improved progression-free survival in the olaparib-treated patients, compared with patients who received the physician’s choice of treatment. In addition, olaparib was better tolerated than the chemotherapy drugs, including capecitabine, vinorelbine or eribulin [47]. Another study on *BRCA1/2*-mutation-associated breast cancer patients, but with a different PARPi, talazoparib, also showed improved progression-free survival. In addition, another phase III randomized trial (OlympiA, NCT02032823) on olaparib demonstrated an improved three-year invasive-free survival of 85.9% vs. 77.1% in the placebo group in the adjuvant setting for patients with a high risk of relapse [48]. Similarly, there are multiple trials that assessed the effect of PARPis as monotherapy or in combination in ovarian cancer. The SOLO2 trial (NCT01874353) investigated the effectiveness of olaparib as a maintenance therapy in sensitive, relapsed high-grade serous ovarian cancer with *BRCA1/2* mutation, which also showed that olaparib had longer progression-free survival, compared to a placebo. As a combination treatment, olaparib was used with bevacizumab as a maintenance treatment for newly diagnosed ovarian cancer patients, and it also showed better progression-free survival vs. bevacizumab alone [49]. Along with monotherapy and combination treatments, PARPis are also used in relapse settings for ovarian cancer (SOLO3 (NCT02282020) and ARIEL 3 (NCT01968213)). Olaparib shows a superior objective response when compared with chemotherapy in patients with *BRCA*-mutated platinum-resistant or partial platinum-sensitive relapsed ovarian cancers [50,51].

Prostate cancer is the fourth most frequent cancer and the fifth leading cause of cancer-related deaths in men globally [46]. Androgen deprivation therapy (ADT) remains the main treatment for advanced prostate cancer. PARPis have been investigated in patients with metastatic castration-resistant prostate cancer (mCRPC) with HR repair defects, demonstrating clinical efficacy for the treatment of these tumors, particularly for those with *BRCA1/2* alterations. In metastatic castration-resistant prostate cancer (mCRPC), *BRCA2* alterations are considerably more prevalent than *BRCA1* alterations, accounting for approximately 14.4% for *BRCA2* and 2.3% for *BRCA1*, comprising both germline and somatic alterations [52,53,54]. Germline *BRCA2* mutations are also a well-established poor prognostic factor in localized and advanced prostate cancer [55,56,57]. Several PARPi-based therapies are currently FDA-approved for molecularly selected patients with mCRPC. Olaparib and rucaparib have monotherapy indications in defined HRR- or *BRCA*-mutated populations, respectively, while approved combination regimens include olaparib with abiraterone and prednisone/prednisolone for *BRCA*-mutated mCRPC, talazoparib with enzalutamide for HRR-mutated mCRPC, and niraparib with abiraterone acetate and prednisone for *BRCA*-mutated mCRPC.

Several clinical studies, including TOPARP-B [58], TRITON2 [52,59], TALAPRO-1 [60], and GALAHAD [61] have, respectively, assessed the efficacy of olaparib, rucaparib, talazoparib, and niraparib in patients with mCRPC who had progressed on prior androgen receptor pathway inhibitors (ARSi) and taxane-based chemotherapy. Across these studies, the most pronounced antitumor activity was generally observed in patients harboring *BRCA1*/2 alterations compared with those harboring other DNA damage repair gene alterations [62], providing important clinical evidence supporting PARP inhibition in molecularly selected mCRPC.

## 4. Innate Resistance to PARP Inhibitors in *BRCA*-Mutant Cancers

Despite the success of PARPis, particularly in *BRCA*-mutant cancers, not all patients respond to PARPis. It has been reported that more than 40% of *BRCA*-mutant ovarian cancer patients fail to benefit from PARPis [63,64]. This is termed innate resistance and refers to resistance prevalent without any prior treatment. Additionally, other studies have suggested that *BRCA1* mutations may be associated with shorter responses to PARPis compared to *BRCA2* mutations, which shows differential sensitivity even between *BRCA*-mutant tumors [65].

Innate resistance mechanisms include up-regulating the p-glycoprotein efflux pump (*P-gp*, *ABCB1* or *MDR1*) [66]. *ABCB1* induction represents a common resistance mechanism in both paclitaxel- and olaparib-resistant ovarian cancer cells. This upregulation is often driven by an innate increased ABCB1 copy number or may result from a chemotherapy-induced chromosomal amplification at 7q11.2–21 representing acquired resistance. Such amplification correlates with elevated P-glycoprotein expression in paclitaxel-resistant cells across multiple cancer types, including ovarian cancer. Drug-resistant phenotypes associated with ABCB1 overexpression have also been supported by studies using P-glycoprotein inhibitors. These findings suggest that certain PARPis are substrates of P-glycoprotein and can be actively effluxed from resistant cells. Notably, resistance to both paclitaxel and olaparib can be reversed when combined with a P-glycoprotein inhibitor. For example, rucaparib is a known P-glycoprotein substrate and is actively effluxed from paclitaxel-resistant cells [67]. Together, these observations suggest that pharmacogenomic markers related to drug efflux, particularly ABCB1, as well as other transporters such as ABCC1 and ABCG2, may play a critical role in predicting sensitivity to PARPis.

Another mechanism of resistance involves the loss of *53BP1*, a gene encoding p53-binding protein 1 [68]. 53BP1 is a major player in DSB repair by promoting NHEJ while suppressing HR [69]. 53BP1 particularly regulates the choice of repair mechanism in BRCA-deficient cells [70], and may therefore influence responses to PARP inhibition. In cells with functional BRCA1, following a DSB, BRCA1 displaces 53BP1, allowing homologous recombination repair (HRR) to proceed. In contrast, when *BRCA1* is mutated and 53BP1 is present, 53BP1 remains bound at DSB sites, suppressing HR and promoting error-prone NHEJ. However, in *BRCA1*-mutant cells lacking 53BP1, downstream HR machinery remains functional, allowing HR to be partially restored. Therefore, the interplay between 53BP1 and BRCA1 determines the balance between HR and NHEJ repair pathways. In a study on *BRCA1*-mutant ovarian cancer, cells with *53BP1* deletion exhibited increased resistance to the PARPi veliparib compared to control cells transduced with an empty vector. These findings are consistent with earlier observations in *BRCA1*-mutant breast cancer and retinal pigment epithelium models, where 53BP1 loss similarly reduced sensitivity to PARP inhibition [71]. Thus, loss of 53BP1 can restore HR capacity in BRCA1-deficient cells and reduce PARPi sensitivity. Depending on whether 53BP1 loss is pre-existing or emerges under therapeutic selection, this mechanism may contribute to either intrinsic or acquired resistance.

## 5. Acquired Resistance to PARP Inhibitors

In addition to some patients being innately resistant to PARPis, others may acquire resistance to PARPis after prolonged exposure to PARPis [72]. A major mechanism of acquired resistance to PARPis involves the restoration of HR repair capability through reversion mutations in *BRCA1/2*. This can happen through a potential second mutation, compensatory mutations, or crossovers typically detected following treatment with chemotherapy, which can partially or fully restore the BRCA1/2 protein function.

Tumor cells may also acquire resistance to PARPis through the upregulation of intracellular NAD+. It has been shown that intracellular NAD+ levels were significantly higher in olaparib-resistant cells than in the non-olaparib-resistant *BRCA1* knockout clone derived from pancreatic cancer cells. Upregulation of intracellular NAD+ levels by the addition of nicotinamide also induced resistance to olaparib and talazoparib in these cells. Since PARPis block PARylation by competing with NAD+ for the catalytic sites of PARP1/2, elevated NAD+ levels, resulting from metabolic adaptation and activation of NAD+ synthesis pathways, may compete with PARPis for binding to the PARP active site, resulting in treatment resistance [73].

PARPi resistance may also be acquired through replication fork protection. “PARP trapping”, resulting from PARPis, causes replication stress and DNA damage. Some cancer cells develop mechanisms to stabilize or protect these replication forks, reducing the effectiveness of PARPis. It has been shown that upregulation of USP1, a deubiquitinase, stabilizes the replication fork, and may thus dampen the response to PARPis and promote survival in BRCA1-deficient tumor cells [74].

Additionally, de novo mutations in alternative DNA repair pathways have shown a strong correlation with resistance to PARPis. For example, an MRE11:p.K464R mutation has been detected during olaparib maintenance therapy in platinum-sensitive relapsed ovarian cancer patients. This mutation enhanced DDR and induced olaparib resistance by promoting MRE11:p.K464R binding to RAD50, a double-strand break repair protein and Ribosomal Protein S3 (RPS3), thus facilitating NHEJ repair in tumor cells. Thus, increased reliance on NHEJ-mediated DNA repair may provide an alternative mechanism for repairing DNA damage and thereby contribute to acquired PARPi resistance [75]. On the other hand, NHEJ can drive PARPi lethality in HR-deficient cells [76]. Hence, the effect of NHEJ on PARPi sensitivity or resistance is actually context-dependent, where error-prone NHEJ can contribute to PARPi lethality in HR-deficient cells, whereas specific adaptations that enhance effective NHEJ-mediated repair, such as the mutation presented above, can provide an alternative repair route and contribute to acquired resistance.

Importantly, most mechanisms of PARPi resistance described above have been established in BRCA1/2-deficient breast, ovarian, prostate, or other cancer models; their prevalence and functional contribution to PARPi resistance in CRC remain largely undefined. Understanding how DNA repair proficiency, metabolic adaptation, and compensatory survival pathways influence PARPi response in CRC may facilitate the development of mechanistic sensitization strategies.

## 6. Colorectal Cancers Are Generally Resistant to PARP Inhibitors

Colorectal cancer (CRC) is the third most common cancer and the second leading cause of cancer-related deaths worldwide, with particularly poor outcomes in patients with metastatic CRC (mCRC), where durable treatment responses remain challenging despite advances in systemic therapy [77]. Current strategies for CRC treatment include surgical excision in combination with chemotherapy and/or targeted therapy depending on the stage of the disease and the mutational profile of the tumor [78]. Treatment relies primarily on fluoropyrimidine-based chemotherapy combined with oxaliplatin or irinotecan, with targeted therapies and immunotherapy selected according to tumor molecular characteristics [79,80,81]. Importantly, treatment response varies substantially by molecular subtype. Patients with mismatch repair-deficient/microsatellite instability-high (dMMR/MSI-H) tumors can achieve substantial benefit from immune checkpoint inhibition, with pembrolizumab demonstrating improved progression-free survival compared with chemotherapy in the first-line setting (16.5 vs. 8.2 months) and objective response rates of approximately 40% or higher reported in selected MSI-H/dMMR cohorts [82,83,84]. In contrast, the majority of CRCs are MSS and generally show limited responses to single-agent immunotherapy, while later-line treatments such as regorafenib, an oral small molecule multi-kinase inhibitor, and trifluridine/tipiracil (TAS-102), an oral chemotherapy, provide limited objective responses and relatively short progression-free survival, highlighting an ongoing need for more effective biomarker-driven therapies [82,83,84].

PARPis could potentially address this unmet need by exploiting defects in DDR pathways and selectively targeting tumors with impaired DNA repair capacity.

Approximately 20% of CRCs harbour somatic alterations in homologous recombination deficiency (HRD)-related genes, including *BRCA1/2* and *ATM.* Moreover, HRD mutational signatures, also known as ‘BRCAness’, have been identified in non-*BRCA1* metastatic CRC [44]. CRC cells with wild-type *TP53* or those that display microsatellite instability (defects in mismatch repair) have shown sensitivity to PARPis, likely due to increased functional alterations in DDR-related genes [85,86]. Despite these indications, PARPis have not demonstrated sufficient clinical efficacy in unselected colorectal cancer, either as monotherapy or when broadly combined with chemotherapy, radiotherapy, or as maintenance therapy, as demonstrated by clinical trials (Table 2). The lack of clinical efficacy of PARPis in unselected CRC is particularly evident from two CRC-specific randomized trials. In the phase III LYNK-003 trial (NCT04456699) in unresectable or metastatic CRC, maintenance olaparib alone or in combination with bevacizumab failed to improve progression-free survival compared with bevacizumab plus a fluoropyrimidine, and the trial was stopped early for futility [87]. Similarly, a randomized phase II trial specifically conducted in previously untreated metastatic CRC (NCT02305758) demonstrated no improvement in progression-free survival with veliparib plus FOLFIRI with or without bevacizumab compared with FOLFIRI with or without bevacizumab alone [88]. However, preliminary observations from selected combination strategies (Table 2), including PARPis plus EGFR inhibition, along with approaches using functional HRD or O6-methylguanine-DNA methyltransferase promoter methylation status (MGMT)-based selection, suggest that PARPis may still have a role in molecularly defined subsets of CRC. Collectively, the current clinical evidence indicates that successful implementation of PARPis in CRC will likely require more precise biological selection than in *BRCA*-associated cancers such as ovarian, breast, pancreatic, or prostate cancer. Importantly, a biomarker or biomarker profile that reliably identifies PARPi-sensitive CRC has not yet been established. Therefore, identifying robust predictive biomarkers and rational combination partners, along with patient stratification, will be critical to improving CRC response to PARPis, as will be discussed in this review.

Although the strategies discussed in this review provide a compelling biological rationale for biomarker-guided PARPi therapy, their levels of clinical development vary considerably, and many of the proposed sensitization approaches remain preclinical or have not been clinically evaluated in CRC. Consequently, prospective validation in molecularly selected CRC populations will be required before these strategies can be translated into routine clinical practice.

## 7. Stratifying CRC Tumors: Biomarkers of PARPi Sensitivity

### 7.1. TP53

**Figure 2:**
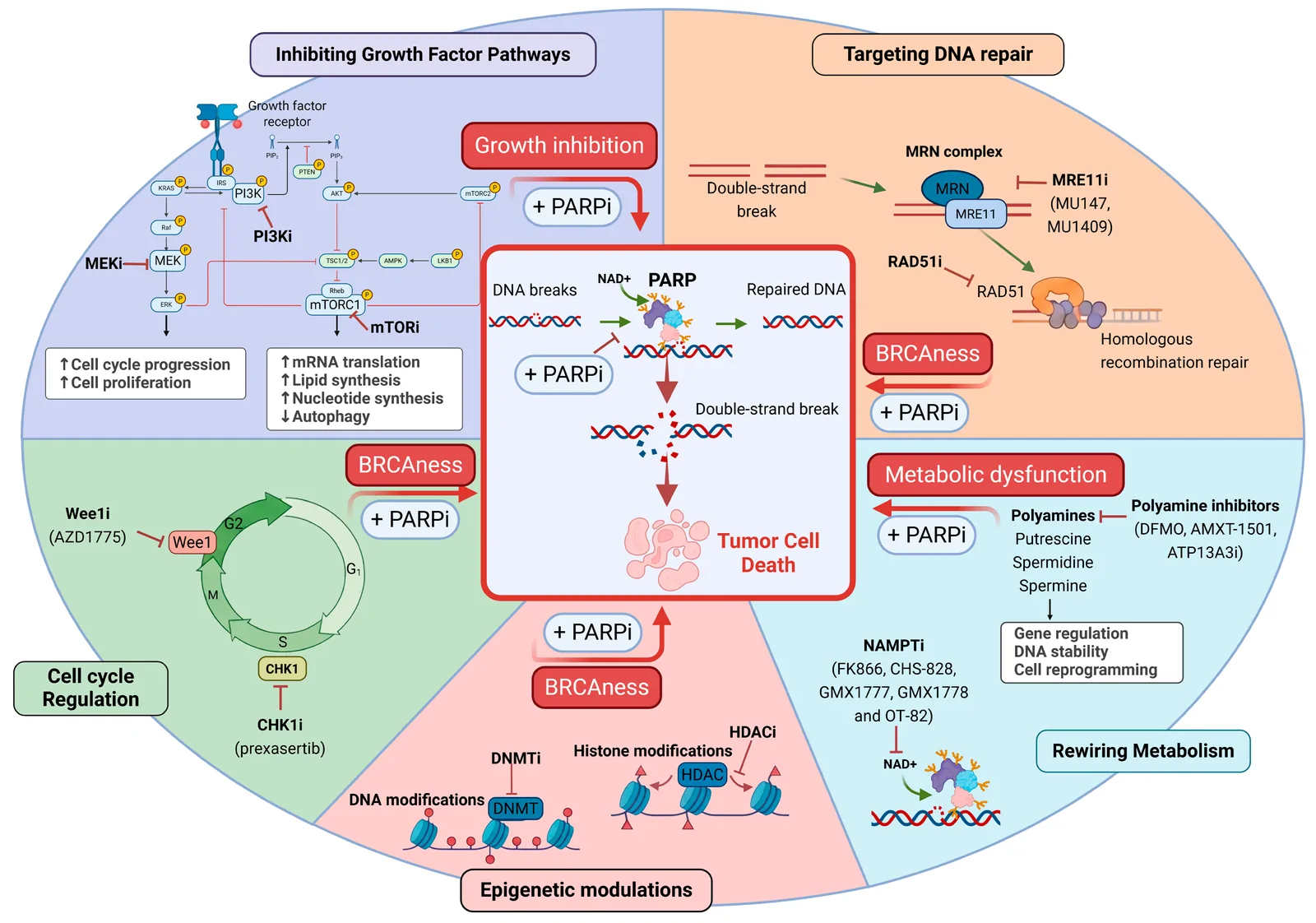
**Mechanisms to sensitize Colorectal Cancer (CRC) cells to PARP inhibitors (PARPis).** Potential therapeutic approaches to sensitize CRC to PARPis by inducing HR defects or exploiting DNA repair vulnerabilities. These include epigenetic modulators, cell-cycle checkpoint inhibitors, growth factor pathway inhibitors, targeting DNA repair, polyamine metabolism inhibitors, and interventions targeting NAD+ metabolism. Blunt arrow (⊣) represents inhibition. Created in BioRender. Naik, A. (2026) Available online: https://BioRender.com/ljq4tl1 (accessed on 10 August 2026).

Wild-type (WT) P53 function has emerged as a candidate preclinical biomarker of PARPi sensitivity in CRC. PARPi sensitivity is strongly associated with *TP53* WT status in CRC cell lines. Gene set expression enrichment analysis (GSEA) showed a significant enrichment with *TP53* WT-associated pathways in PARPi-sensitive CRC samples. Further supporting this, transcriptional activity downstream of *TP53* is a primary response to PARP inhibition in CRC cell lines. GSEA of the most upregulated genes (PARPi treatment versus control) in the two CRC-sensitive cell lines showed that DNA damage repair, and more specifically *TP53* activity, was among the primary response mechanisms. Notably, the five most upregulated genes (*GDF15*, *PLK2*, *MDM2*, *TP53INP1*, *RRM2B*) were all known transcriptional targets of *TP53* and were upregulated exclusively in the sensitive cell lines. Hence, WT *TP53* has emerged as the strongest preclinical biomarker associated with PARPi sensitivity in preclinical CRC models [86]. In the high-throughput screen by Smeby et al. [86], eight CRC cell lines were classified as PARPi-sensitive, of which six (75%) harbored wild-type *TP53*, while two (HROC87 and COLO205) carried *TP53* mutations. These findings indicate that although wild-type *TP53* is enriched among PARPi-sensitive CRC models, *TP53* status alone is insufficient to fully explain treatment response. This observation is consistent with subsequent mechanistic studies demonstrating that p53-mediated regulation of DNA damage response pathways, including suppression of RAD51, may contribute to PARPi sensitivity. Although these findings are based primarily on preclinical models, they support further evaluation of *TP53* as part of a broader biomarker panel for patient stratification in CRC (Figure 2) [103]. A recent study further demonstrated that PARP1 regulates the cancer stem cell phenotype in CRC in a p53-dependent manner, reinforcing the biological interplay between p53 and PARP signaling in CRC, although PARPi response was not directly investigated [104].

### 7.2. RAD51

Beyond *TP53* status, RAD51 has emerged as a complementary biomarker and therapeutic target that may further refine prediction of PARPi sensitivity in CRC. RAD51 is a crucial player in HR that facilitates DSB repair by binding to DNA and mediating homologous pairing and strand exchange. Up-regulation of *RAD51* reduces sensitivity to excessive DNA damage in cancer cells and represents a compensatory pathway for other deficient repair mechanisms (Figure 2). The relationship between *TP53* and RAD51 is particularly relevant in CRC. *TP53*-dependent RAD51 downregulation has been reported in CRC cell lines. In cells with WT *TP53*, RAD51 foci formation after DSBs is inhibited. However, when *TP53* is mutated, RAD51 mRNA, protein expression and foci formation are no longer repressed. This suggests that transcriptional repression of RAD51 by p53 may help regulate HR, whereas impaired RAD51 repression by mutant p53 may contribute to malignant transformation [105]. Because *TP53* mutations are common in tumor cells, RAD51 is often overexpressed, thus increasing the resistance to DNA damage. Therefore, wild-type p53-mediated suppression of *RAD51* is a possible mechanism by which PARPis exhibit their activity [86]. This makes RAD51 a potentially CRC-relevant biomarker, particularly in tumors with wild-type *TP53*. Supporting this, functional analysis comparing olaparib-sensitive and olaparib-resistant CRC cell lines showed that the number of RAD51 foci-positive nuclei increased in all olaparib-resistant cells, but not in the sensitive cells. Future studies should evaluate the feasibility of detecting RAD51 foci using tissue-based immunofluorescence assays in clinical CRC specimens as a predictive biomarker for PARPi sensitivity [106]. It is also worth noting that while p53-mediated RAD51 repression provides a mechanistic rationale for differences in HR competency, RAD51 functional status may represent the more clinically relevant biomarker for predicting PARPi sensitivity [105].

### 7.3. MRE11

Another relevant biomarker is the DSB repair gene *MRE11* [75,107]. Defective DNA mismatch repair (MMR) generates mutations at repetitive DNA sequences, including microsatellite tracts in *MRE11*. This has been studied in CRCs displaying microsatellite instability (MSI), which occurs in approximately 15% of CRCs, and results from germline or epigenetic inactivation of MMR genes. MSI is associated with mutations in more than 30 genes containing microsatellite repeats, including genes encoding DSB repair proteins involved in the HR pathway, such as *MRE11* and *RAD50*. The mutational status of *MRE11* was assessed in a panel of 17 CRC cell lines and 46 primary tumors, revealing a strong correlation with MSI status in both cell lines and tumors. The activity of a PARP1 inhibitor, ABT-888, was tested on two models: one utilized the knockdown of *MRE11* in a WT and microsatellite-stable (MSS) CRC cell line (SW-480), and in the other, cell lines were transfected with mutant *MRE11.* The PARPis had preferential cytotoxicity in the MSI cell lines harboring mutations in *MRE11* compared to WT cell lines and MSS cell lines. Together, these findings suggest that functionally consequential MRE11 defects, rather than *MRE11* mutation status alone, may contribute to PARPi sensitivity in MSI CRC (Figure 2). Accordingly, the predictive relevance of MRE11 alterations will require assessment of their functional consequences rather than classification based solely on mutation status.

Although *TP53*, RAD51, and *MRE11* represent promising candidate biomarkers of PARPi sensitivity, their predictive value, individually or as part of integrated genomic and functional HRD assessments, requires prospective validation in independent clinical cohorts before implementation in patient stratification. Additionally, the presence of such alterations does not necessarily indicate functional HR deficiency or predict sensitivity to PARP inhibition. Importantly, the functional significance of HR-associated alterations depends on their pathogenicity, allelic status, and genetic context, including whether an alteration is monoallelic or biallelic, germline or somatic, and accompanied by loss of heterozygosity [108]. Beyond individual gene alterations, HRD can also be assessed using genomic-scarring measures, mutational signatures, and functional assays, which provide complementary information on homologous recombination competency [109]. Therefore, gene-level alterations should be viewed as potential indicators of impaired DNA repair rather than definitive evidence of functional HRD unless supported by additional genomic or functional evidence [108,109]. For patient selection, a more reliable classification of HRD should consider the nature and pathogenicity of the underlying alteration, evidence of biallelic inactivation or loss of heterozygosity, and/or independent genomic or functional evidence of impaired homologous recombination [108,109].

## 8. Enhancing PARPi Sensitivity in CRC

Here we discuss promising mechanistic combinatorial strategies to impair DNA repair capacity, alter cellular metabolism, or induce a BRCAness-like phenotype, thereby increasing dependence on PARP-mediated repair pathways and enhancing sensitivity to PARPi-induced synthetic lethality (Figure 2). Unless otherwise stated, the majority of these strategies are supported by mechanistic and preclinical evidence derived from CRC models or other solid tumors. Although they provide a strong biological rationale for improving PARPi efficacy, relatively few have progressed to CRC-specific clinical evaluation.

### 8.1. Immune Checkpoint Blockade

Immune checkpoint inhibitors are the current standard of care for patients with deficient mismatch repair (dMMR)/microsatellite instability-high (MSI-H) tumors, where they have demonstrated remarkable and durable clinical benefit [110]. However, a subset of patients with MSI-H/dMMR CRC fails to respond or eventually develops resistance to immune checkpoint blockade, highlighting the need for additional therapeutic strategies [111]. Hence, emerging evidence suggests that PARP inhibition may enhance antitumor immunity and provide a rationale for combination strategies with immunotherapy. In CRC, the relationship between MSI status and PARPi sensitivity remains controversial. Some studies have proposed MSI as a predictive biomarker of PARPi response [112] whereas others have reported no significant difference in niraparib sensitivity between MSI and MSS CRC cell lines, suggesting that MSI status alone may be insufficient to predict PARPi responsiveness [113]. Consequently, further mechanistic and translational studies are required to define the role of MSI as a biomarker for PARPi-based therapies.

In other cancers, combining PARPis and immune checkpoint inhibitors is under investigation, and a few clinical trials exist for treating solid tumors, particularly in ovarian, breast, and prostate cancers. For example, the MEDIOLA trial, an open-label, multicentre, phase 1/2 basket study evaluated the combination of olaparib and the PD-L1 inhibitor durvalumab in patients with germline *BRCA*-mutated metastatic breast cancer. The combination demonstrated promising antitumor activity. Similarly, the TOPACIO trial reported promising activity for the combination of niraparib and the PD-1 inhibitor pembrolizumab in patients with advanced or metastatic triple-negative breast cancer [114,115]. Although these findings cannot be directly extrapolated to CRC, they provide a strong biological rationale for investigating PARPi–immunotherapy combinations in molecularly selected CRC populations, particularly MSI-H/dMMR tumors. Prospective CRC-specific preclinical and clinical studies will be required to determine whether these combinations improve therapeutic efficacy and to identify the patient populations most likely to benefit.

### 8.2. Epigenetic Modulation

BRCAness can be induced by the modulation of epigenetic pathways by targeting DNA methyltransferases (DNMT) or histone deacetylases (HDACs). DNMT inhibitors (DNMTi), such as decitabine and 5-azacytidine, trap DNMT on DNA, disrupting methylation and enhancing tumor cell death. Studies have shown the synergy between DNMTi and PARPi efficacy, as both DNMT1 and PARP1 localize to DNA damage sites. DNMTi can inhibit DNA repair mechanisms, resulting in a BRCAness phenotype, along with increased cytotoxicity that has been reported in several human cancer cell lines and murine xenograft models [116,117]. On the other hand, HDACs remove acetyl groups from histones, leading to chromatin condensation and the repression of genes involved in tumor suppression. HDAC inhibitors (HDACi), such as vorinostat and depsipeptide, which are FDA-approved [118], can sensitize cells to PARPis as they disrupt DNA repair by altering chromatin structure and reducing the expression of HR proteins, including BRCA1/2. Combination therapies including HDACi and PARPis enhance PARP trapping and DNA damage in acute myeloid leukemia (AML) [119], and a triple combination with DNMTi in leukemia and lymphoma patients has been reported to further increase DNA damage, ROS production, and apoptosis, leading to tumor cell death [120]. This pathway may also be relevant to CRC, in which DNMT1 alterations and aberrant DNA methylation have been reported [121]. However, whether these features predict sensitivity to combined DNMT and PARP inhibition in CRC remains unknown, although promising pre-clinical evidence in a mouse CRC model indicated an inhibitory effect with a treatment combination of a DNMTi and mTORi [122].

The modulation of epigenetic pathways in combination with PARPis is currently in a phase II clinical trial in luminal advanced breast cancer (clinicaltrials.gov, NCT04355858, [123]), including an epigenetic cohort combining the EZH2 inhibitor SHR2554 with the PARPi SHR3162. EZH2 is a histone methyltransferase involved in the regulation of DNA-damage repair and has functional interactions with PARP1. Following DNA damage, PARP1 can PARylate EZH2 and suppress its histone methyltransferase activity [124]. Importantly, EZH2 inhibition has been shown to sensitize HR-proficient tumors to PARPis in a context-dependent manner. In CARM1-high epithelial ovarian cancer, EZH2 inhibition upregulates MAD2L2, promotes NHEJ and reduces DNA end resection, resulting in increased chromosomal abnormalities and mitotic catastrophe following PARP inhibition [125]. Thus, targeting EZH2 may extend PARPi sensitivity to HR-proficient tumors by altering DNA-repair pathway choice toward NHEJ.

Alternative epigenetic strategies may involve modulation of H3K4 methylation and its effects on RIF1 recruitment to DSBs. SETD1A-dependent H3K4 methylation promotes RIF1 recruitment to DSBs and facilitates NHEJ, whereas loss of SETD1A-dependent H3K4 methylation increases end resection, restores HR in BRCA1-deficient cells, and can confer PARPi resistance [126]. Thus, pharmacological manipulation of H3K4 methylation or demethylation pathways may influence PARPi response, although the therapeutic implications of these approaches remain context-dependent and require further investigation in CRC.

### 8.3. Modulating Cell Cycle Regulators

Several cell cycle checkpoint proteins directly interact with DSB repair proteins, and therefore targeting these may offer a potential for inducing BRCAness. For example, the inhibition of CDK12 sensitizes *BRCA*-wild type triple negative breast cancer (TNBC) cells and patient-derived xenografts (PDX) to PARPis as CDK12/13 functions as an RNA polymerase II C-terminal domain (CTD) kinase that stabilizes long DNA repair gene transcripts and regulates the transcription of numerous DNA repair genes, particularly those involved in HR [127]. Additionally, CDK9 inhibition in combination with olaparib significantly suppresses cell viability and colony formation, along with inducing apoptosis in BRCA1-proficient ovarian cancer cells, and also remarkably reduces tumor growth in mouse xenograft models [128]. Clinical trials are still pending for CDK inhibitors; however, multiple pre-clinical studies have established their beneficial role in combination with PARPis.

Clinical trials investigating combinations of ATR inhibitors (ATRis), such as AZD6738 and VX-970, with PARPis in *BRCA*-mutated cancers are currently underway [129]. ATR is crucial in the DDR, particularly during S phase, where it detects stalled replication forks by binding to replication protein A-coated single-stranded DNA. ATR signaling supports RAD51 recruitment and repair at DSBs. In *BRCA1*-mutated cells, ATR restores HR by facilitating RAD51 loading, potentially reversing BRCA1-associated repair defects and inducing PARPi resistance. Several clinical trials combining the ATRis AZD6738 and olaparib are underway in PARPi-naïve and PARPi-treated cancers (clinicaltrials.gov, NCT02264678) [130,131,132].

CHK1 and Wee1 inhibitors may also improve sensitivity to PARPis. A combination of prexasertib, a Chk1/Chk2 inhibitor, and olaparib resulted in synergistic cytotoxic effects against *BRCA* WT high-grade serous ovarian cancer cells [133]. However, unlike ATR inhibitors, CHK1 inhibition is less impactful for PARPi sensitization as it only causes minimal DNA damage accumulation [134,135] and therefore its success in clinical trials has been limited, with only one phase I trial reported for advanced solid tumors (clinicaltrials.gov, NCT03057145). Wee1 is a Ser/Thr kinase that phosphorylates CDK1 at the inhibitory Tyr15 residue, thereby suppressing CDK1 activity to regulate G2 phase progression, and it sustains ATR and CHK1 activity during the DDR. Its inhibition activates CDK1, phosphorylating BRCA2 and limiting HR. Wee1 also protects replication forks by negatively regulating the endonuclease MUS81. Consequently, Wee1 inhibitors, such as AZD1775, can slow replication fork progression, activate the DDR, and enhance sensitivity to olaparib in various cancers, prompting ongoing clinical trials [129,136]. Phase I/II clinical trials examining olaparib in combination with inhibitors of Wee1 (AZD1775) (Clinicaltrials.gov, NCT02511795, NCT03579316 and NCT04197713) are underway.

Although CHK1 and WEE1 are not commonly mutated in CRC, their functional importance is enhanced in CRC tumors harboring *TP53* or *KRAS* mutations. In a study, the WEE1 inhibitor adavosertib (AZD1775) was tested on multiple CRC cell lines, and it was reported to suppress cell proliferation in a concentration-dependent manner in all CRC cell lines. The inhibitor also caused an accumulation of cells in the G2/M phase and elevated apoptosis levels, particularly in CRC cell lines carrying *TP53* mutations, while having little to no effect on most *TP53* wild-type cell lines. In an orthotopic CRC mouse model, treatment with the WEE1 inhibitor led to a significant reduction in tumor volume compared to controls. Overall, these findings suggest that WEE1 inhibitors show antitumor activity in CRC, especially in tumors with *TP53* mutations [137]. Therefore, these inhibitors may represent a strategy to induce a “BRCAness” phenotype and enhance sensitivity to PARPis when used in combination in CRC.

### 8.4. Growth Factor-Related Pathways

Receptor tyrosine kinases (RTKs), such as c-MET and VEGFR, are frequently mutated, and constitutively activated in cancer, and thus can be targeted in combination with PARPis [129]. C-MET is an RTK that can promote cellular survival under oxidative stress through anti-apoptotic and antioxidant signaling [138]. It has been shown to directly associate with and phosphorylate PARP1 at Tyr907 within the catalytic domain, increasing PARP1 enzymatic activity and reducing PARPi binding, thereby promoting PARPi resistance. PARP1 pTyr907 may therefore represent a potential predictive biomarker of c-MET-mediated PARPi resistance [139]. Accordingly, inhibition of c-MET reduces PARP1 Tyr907 phosphorylation, sensitizing cells to PARPis with synergistic antitumor activity demonstrated in preclinical TNBC and lung cancer models [139], in addition to similar synergistic effects demonstrated in HGSOC models [140]. Notably, hyperactivation of c-MET and EGFR signaling has been observed in *BRCA1*-mutant TNBC cells with acquired PARPi resistance, whereas combined inhibition of c-MET and EGFR can restore PARPi sensitivity [141].

Moreover, VEGFR inhibition leads to tumor hypoxia, which is linked to HR defects via the downregulation of *BRCA1*, *BRCA2*, and *RAD51*. In CRC, a recent phase II clinical trial (NIPAVect, NCT03983993) evaluated the combination of the PARPi niraparib with the EGFR inhibitor panitumumab in patients with advanced or metastatic RAS wild-type CRC. The combination demonstrated an acceptable safety profile and encouraging preliminary antitumor activity, providing initial clinical evidence supporting PARPi-based combination strategies in CRC [95]. In addition, a phase II clinical trial in platinum-sensitive HGSOC indicated that the combination of VEGFR inhibition and PARPis prolongs progression-free survival over single-agent treatment. Several early-stage clinical trials combining the VEGFR inhibitor cediranib with olaparib are currently underway (clinicaltrials.gov, NCT01116648) [142].

Mitogen-activated protein kinase kinase (MEK) inhibitors (MEKis) have also been proposed for use in combination with PARPis. MEKis inhibit double-strand break repair, thereby sensitizing *KRAS*-mutant breast, ovarian and endometrial cancers to talazoparib. This effect is accompanied by increased *PARP1* expression, which enhances accumulation of cytotoxic PARP-trapping lesions. Similar observations have been made in pancreas and ovarian cancer models [143], suggesting a combinatorial role for PARPis and MEKis in the treatment of PARPi-resistant and/or *KRAS*-mutant tumors, thus prompting clinical testing in a phase I/II trial (clinicaltrials.gov, NCT03162627).

Similarly, phosphoinositide 3-kinase (PI3K) inhibitors can also be used to induce DSB repair defects. In both in vitro and in vivo models of BRCA-proficient TNBC, treatment with the PI3Ki BKM120 induced *BRCA1/2* downregulation [144]. Comparable effects were observed with the mTOR inhibitors everolimus or KU0063794 [145] and the dual PI3K/mTOR inhibitor GDC-0980, thus impairing HR and sensitizing tumors to PARPis [146]. PI3K pathway inhibition has also progressed to clinical evaluation in combination with PARPis, although not specifically in CRC. Early-phase studies of olaparib with the PI3K inhibitors buparlisib or alpelisib (NCT01623349) demonstrated the feasibility and preliminary antitumor activity of this approach in breast and ovarian cancers [147]. However, clinical efficacy appears context-dependent, as the combination of talazoparib with the PI3K/mTOR inhibitor gedatolisib (NCT03911973) failed to meet its prespecified efficacy threshold in advanced breast cancer [148]. Phase I/II clinical trials examining olaparib in combination with inhibitors of AKT (AZD5363) or mTOR (AZD2014) (clinicaltrials.gov, NCT02208375) are underway. NCT02208375 is evaluating olaparib in combination with the AKT inhibitor capivasertib (AZD5363) or the dual mTORC1/2 inhibitor vistusertib (AZD2014) in recurrent ovarian, endometrial, and triple-negative breast cancers, further supporting clinical investigation of PARPi combinations targeting the PI3K/AKT/mTOR axis. In CRC, combined treatment with the mTOR inhibitor rapamycin (RPM) and the DNA methyltransferase inhibitor 5-aza-deoxycytidine (AZA) suppressed HCT116 cell growth, triggered apoptosis, caused cell cycle arrest, and reduced both tumor incidence and volume in mouse models. This dual therapy also decreased phosphorylation of key proteins in the mTOR signaling pathway, showing stronger effects than either drug alone [122].

### 8.5. Targeting DNA Repair

Beyond their role as predictive biomarkers, MRE11 and RAD51 are increasingly being investigated as therapeutic targets for overcoming PARPi resistance. MRE11 inhibitors have already been developed, with mirin being the first inhibitor identified. Pharmacological inhibition of MRE11 using mirin induces synthetic lethality in BRCA2-deficient ovarian cancer cells, HeLa cells, and 3D spheroid models compared to BRCA2-proficient controls [149], suggesting that further impairment of homologous recombination repair may enhance susceptibility to PARPis. More recently, the next-generation inhibitors MU147 and MU1409 have demonstrated greater specificity and potency than mirin while selectively inhibiting MRE11 nuclease activity without impairing ATM activation [150].

Similar to MRE11, RAD51 has emerged as a potentially therapeutically actionable component of the homologous recombination machinery. Several direct RAD51 inhibitors have been developed preclinically, including RI-1 and its reversible analogue RI-2, which interfere with RAD51 oligomerization, B02, which disrupts RAD51–DNA binding and strand-exchange activity, and IBR2, which disrupts RAD51 multimerization and promotes its degradation. Amuvatinib, a multitargeted tyrosine-kinase inhibitor, has also been shown to indirectly modulate RAD51 expression. However, its phase II evaluation with platinum–etoposide in platinum-refractory small-cell lung cancer (NCT01357395) did not meet its prespecified efficacy threshold, and the study is now completed [151]. Antibody-based strategies have also been investigated, including the cell-penetrating 3E10 autoantibody, which directly binds RAD51 and inhibits homology-directed repair, and Fab-F2-iPTD, which binds RAD51 and inhibits RAD51–single-stranded DNA interactions. More recently, humanized 3E10 variants have retained RAD51-targeting and homology-directed repair inhibitory activity while demonstrating tumor-targeting properties, although these approaches remain preclinical [152,153].

CYT-0851 was initially developed as an inhibitor of RAD51-mediated homologous recombination and entered phase I/II evaluation in advanced hematological malignancies and solid tumors (NCT03997968) [154]. Subsequent mechanistic work, however, indicated that CYT-0851 primarily acts as a monocarboxylate transporter inhibitor rather than as a direct RAD51 inhibitor. Development of CYT-0851 was discontinued in 2023 after its overall clinical efficacy data did not meet the developer’s criteria for advancement. Nevertheless, direct targeting of RAD51 remains an active area of preclinical drug development, with newer approaches including structure-guided inhibitors and, more recently, the development of proteolysis-targeting chimeras (PROTACs) directed to RAD51, based on the RAD51-targeting small-molecule inhibitors RI-1 and RI-2 [155]. Collectively, these findings support RAD51 as a biologically compelling, but as yet clinically unvalidated, therapeutic target for disrupting HR and potentially enhancing sensitivity to PARP inhibition.

Targeting MRE11 may also influence PARPi response through effects on HR and replication-fork stability; however, its role is context-dependent, as MRE11 loss can increase PARPi sensitivity in some settings whereas attenuation of MRE11-mediated replication-fork degradation can contribute to PARPi resistance in BRCA-deficient cells [156,157]. Thus, although modulation of MRE11- and RAD51-associated repair pathways represents a potential strategy for overcoming PARPi resistance, further mechanistic and clinical validation is required, particularly in CRC.

### 8.6. Pharmacological Ascorbate

High doses of vitamin C (ascorbate) have been evaluated as an anti-cancer therapy in a range of malignancies, such as ovarian cancer, pancreatic cancer, NSCLC, and glioblastoma. High-dose ascorbate generates hydrogen peroxide, leading to oxidative DNA damage that activates PARP1, resulting in depletion of intracellular NAD+, subsequent ATP depletion, and ultimately cell death. Concurrent PARP inhibition is predicted to exacerbate this damage by impairing base excision repair and promoting PARP trapping on DNA, thereby overwhelming the DNA repair capacity of tumor cells linked to the ascorbate-induced downregulation of BRCA1, BRCA2, and RAD51 [158,159]. These observations provide a strong biological rationale for evaluating this combination in future CRC preclinical studies and biomarker-driven clinical trials. To date, no registered clinical trials have evaluated the combination of pharmacological ascorbate and PARPis in CRC. Nevertheless, high-dose intravenous ascorbate has demonstrated an acceptable safety profile in patients with metastatic CRC when administered in combination with standard chemotherapy regimens (mFOLFOX6 or FOLFIRI), establishing a recommended dose of 1.5 g/kg/day [160]. The rationale for combining pharmacological ascorbate with PARP inhibition is therefore based on mechanistic and preclinical evidence rather than clinical efficacy data.

### 8.7. Polyamine Inhibition

Polyamines, which include the aliphatic cations putrescine, spermidine, and spermine, are small organic molecules integral to various biological processes such as cell growth, differentiation, and DNA repair mechanisms [161,162]. Studies have shown that polyamines can influence DNA damage responses, including promotion of homology-directed DNA repair [163,164]. When polyamine levels are depleted, cells become more sensitive to DNA damage and accumulate strand breaks [162], suggesting that cancer cells may be especially dependent on polyamine metabolism to maintain DNA integrity under stress.

This dependence has made polyamine metabolism an increasingly attractive target in cancer therapy. One promising approach involves using polyamine synthesis inhibitors like α-difluoromethylornithine (DFMO), which targets ODC1, the enzyme responsible for the first step in polyamine biosynthesis [165,166]. In cancers that heavily rely on polyamines for survival, DFMO treatment can lead to reduced proliferation and, in some contexts induce cell death. For instance, in diffuse intrinsic pontine glioma (DIPG), a highly aggressive pediatric brain tumor, DFMO treatment was shown to suppress polyamine synthesis. By combining DFMO with a polyamine transport inhibitor, AMXT 1501, researchers were able to shut down both the synthesis and uptake pathways, significantly reducing tumor growth and improving survival in mouse models [167]. A similar pattern was observed in high-risk neuroblastoma, where Azfar et al. identified ATP13A3 as the main transporter responsible for DFMO-induced polyamine uptake. Inhibiting ATP13A3 enhanced the effect of DFMO, suggesting that dual targeting of synthesis and transport could be an effective therapeutic strategy [168].

In CRC, polyamine metabolism is often dysregulated and has been associated with tumor growth and malignant phenotypes [169,170]. CRC-specific studies have also shown that targeting polyamine-related pathways can affect tumor growth and malignant phenotypes. For example, Zhang et al. [170] demonstrated that apigenin disrupts a HIF-1α/SMOX-positive feedback loop in CRC, reducing proliferation, migration, and tumor growth, while polyamine supplementation was able to rescue apigenin-induced cytotoxicity. These findings support polyamine metabolism as a CRC-relevant metabolic vulnerability, although this study did not directly examine PARP inhibition or canonical DNA damage repair.

The potential connection between polyamine metabolism and PARPis therefore remains primarily mechanistic. Polyamine perturbation has been associated with altered DNA damage responses, cellular stress, and metabolic/redox changes [163,167,171]. In parallel, PARP activity is closely linked to NAD+ metabolism, DNA damage signaling, and cellular energy homeostasis, providing a potential mechanistic bridge between polyamine metabolism and PARP-targeted therapy. Nevertheless, direct CRC-specific preclinical or clinical evidence demonstrating synergy between polyamine-targeting therapies and PARPis remains limited.

Collectively, current evidence supports polyamine metabolism as a biologically relevant vulnerability in CRC and provides a mechanistic rationale for investigating its combination with PARP inhibition. However, the proposed combination remains largely unvalidated in CRC, and dedicated preclinical studies are needed to determine whether polyamine inhibition can directly enhance PARPi sensitivity or overcome PARPi resistance.

### 8.8. Targeting NAD+ Metabolism

Targeting NAD+ metabolism to sensitize tumors to PARPis has emerged as a promising strategy to amplify DNA damage-associated stress and further restrict DNA repair capacity in cancer cells. PARP-1 is a major NAD+-consuming enzyme that catalyzes poly(ADP-ribosyl)ation during DNA repair. Consequently, sustained PARP activation can rapidly deplete intracellular NAD+ pools, disrupt glycolysis and mitochondrial ATP production, and create energetic vulnerabilities that may be further exploited through therapeutic interventions targeting NAD+ metabolism [172,173].

Building on this metabolic dependence, strategies that further limit NAD+ availability, most notably through inhibition of nicotinamide phosphoribosyltransferase (NAMPT), the rate-limiting enzyme in the NAD+ salvage pathway, have demonstrated strong preclinical synergy with PARPis. NAMPT inhibitors, including FK866, CHS-828, GMX1777, GMX1778, and OT-82, deplete intracellular NAD+ levels and sensitize tumor cells to PARPis by enhancing replication stress, DNA damage accumulation, and apoptosis [174,175,176,177]. In Ewing sarcoma, combined PARP and NAMPT inhibition produced synergistic tumor cell killing associated with NAD+ depletion, increased DNA damage, and apoptosis, and enhanced antitumor activity in vivo [177]. Importantly, the extent of sensitization appears to vary across tumor types, likely reflecting differences in alternative NAD+ biosynthetic pathways and tumor-specific dependence on NAMPT-mediated NAD+ salvage. In parallel, NAD+ is consumed by multiple enzyme families, including PARPs, sirtuins, and NADases such as CD38/CD157, creating competition for compartmentalized NAD+ pools that influences cellular metabolism and redox homeostasis [174,178,179]. At the same time, PARPis themselves may alter NAD+ utilization and increase SIRT1 activity, promoting mitochondrial biogenesis and oxidative metabolism [178,180,181]. Such metabolic adaptations may influence cellular survival and treatment response, although their contribution to PARPi resistance remains context-dependent. These findings underscore the context-dependent nature of NAD+-targeted therapeutic strategies and the importance of identifying tumor-specific metabolic dependencies, particularly in the context of acquired PARPi resistance, where metabolic adaptation and maintenance of NAD+ homeostasis may support tumor cell survival despite continued PARP inhibition. However, direct evidence supporting NAD+-targeting strategies in combination with PARPis in CRC remains limited. Although NAMPT inhibition and NAD+ modulation have demonstrated PARPi sensitization in other tumor models, these findings have not been explored in CRC.

Collectively, current evidence suggests that tumors with strong mitochondrial reliance or distinct NAD+-related metabolic vulnerabilities may be particularly susceptible to NAD+-limiting approaches in combination with PARPis, whereas metabolically flexible tumors may exhibit more variable responses [159,172,173]. However, given the central role of NAD+ in normal tissue homeostasis, systemic NAD+ depletion also raises concerns regarding toxicity, impaired redox regulation, and effects on non-malignant DNA repair processes. As a result, current approaches increasingly focus on tumor-selective targeting strategies, such as exploiting elevated NAMPT dependence or developing targeted delivery systems, to maximize therapeutic efficacy while minimizing adverse effects [175,182,183]. Overall, combining PARPis with interventions targeting NAD+ metabolism represents a mechanistically compelling strategy to overcome therapeutic resistance and expand the clinical utility of PARP inhibition in CRC, although successful clinical translation will require validation in CRC-specific pre-clinical and clinical studies, careful consideration of tissue specificity, NAD+/NADH compartmentalization, and tumor metabolic context.

## 9. Conclusions

**Figure 3:**
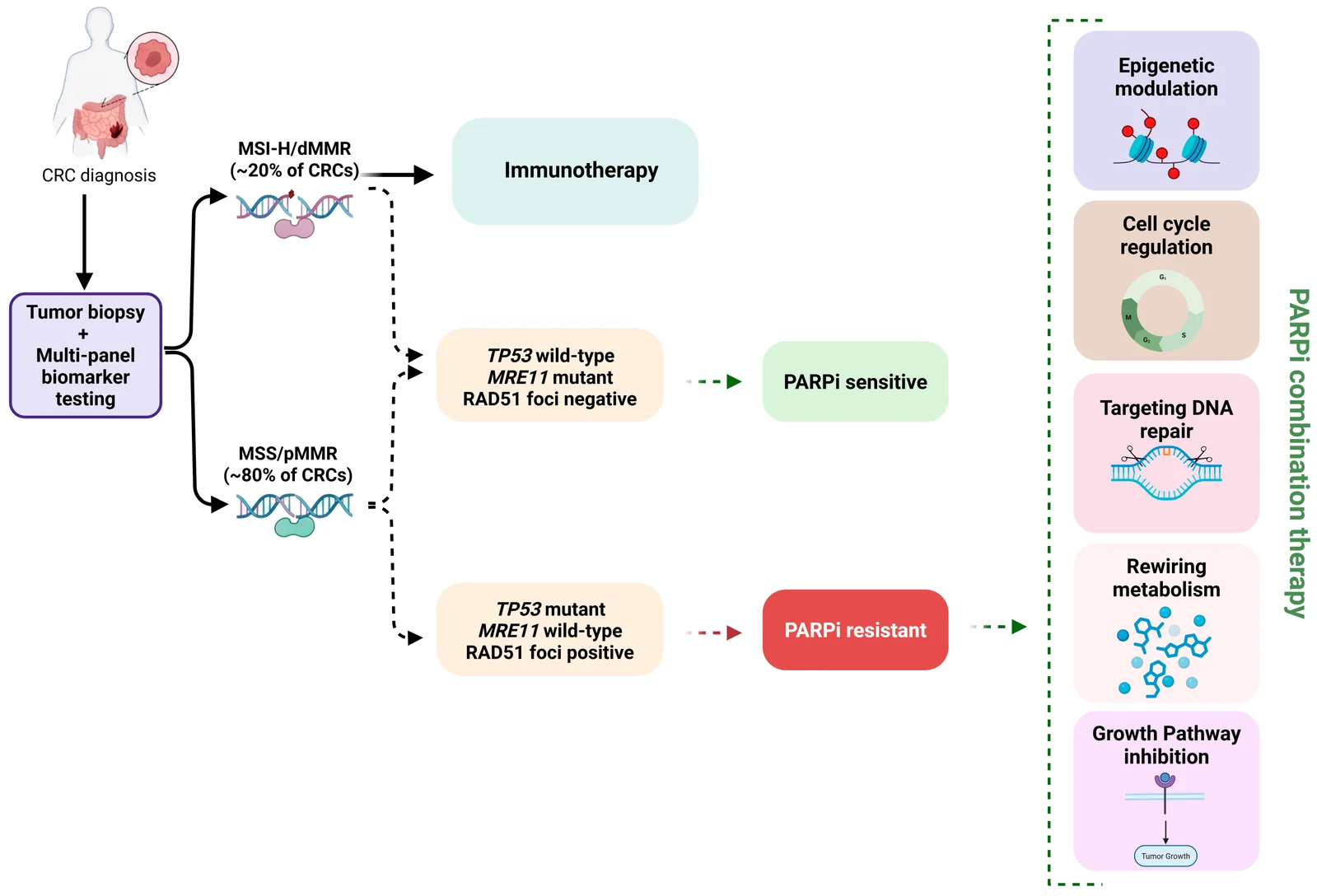
**Proposed molecular stratification framework for biomarker-guided PARP inhibitor (PARPi) therapy in colorectal cancer (CRC).** Based on mechanistic evidence from multiple tumor types and the limited CRC-specific literature, this proposed conceptual framework illustrates a potential strategy for molecular stratification of CRC patients. Tumors are first classified according to microsatellite instability-high (MSI-H)/mismatch repair-deficient (dMMR) or microsatellite-stable (MSS)/mismatch repair-proficient (pMMR) status, followed by assessment of candidate biomarkers including *TP53*, RAD51, and *MRE11*, which may influence PARPi sensitivity. The resulting molecular profile may guide the selection of rational combination strategies targeting epigenetic regulation, cell-cycle checkpoints, DNA repair pathways, growth factor signaling, and metabolic vulnerabilities to overcome PARPi resistance. This framework is intended as a hypothesis-generating model to inform future preclinical studies and biomarker-driven clinical trials in CRC. Solid black arrows indicate literature evidence-based stratification, dashed black arrows indicate proposed candidate biomarker-based stratification, dashed red arrows indicate proposed classification of PARPi-resistant CRC, dashed green arrows indicate proposed classification for PARPi-sensitive CRC and combination strategies to sensitize PARPi-resistant tumors. Created in BioRender. Masood, E. (2026) https://BioRender.com/zzsrjbp (accessed on 10 August 2026).

Although PARPis have demonstrated remarkable clinical success in *BRCA1/2*-mutant cancers, their efficacy in CRC remains limited. Clinical studies in broadly selected CRC populations have not demonstrated sufficient benefit. Current pre-clinical evidence suggests that a subset of CRCs possess molecular or functional characteristics that may confer PARPi sensitivity, including functional defects in DDR pathways, microsatellite instability, wild-type *TP53* status, and functional alterations in RAD51 and MRE11. Among these candidates, wild-type *TP53* currently has the strongest preclinical evidence as a candidate biomarker associated with PARPi sensitivity associated with PARPi sensitivity in CRC, while MRE11 may be particularly informative in microsatellite instability tumors and RAD51 represents a promising functional biomarker and therapeutic target. While wild-type *TP53* is enriched among PARPi-sensitive CRC models, *TP53* status alone is unlikely to fully predict treatment response, highlighting the need for integrated biomarker panels incorporating complementary molecular features (Figure 3). Accordingly, biomarker panels integrating *TP53*, RAD51, *MRE11*, microsatellite instability, gene-level DDR alterations, HRD-associated genomic signatures, and functional measures of HR competency warrant evaluation as an integrated approach to patient stratification. Consequently, improved biomarker-based patient stratification is likely to be essential for successful implementation of PARPi-based therapies in CRC [184].

Beyond patient selection, multiple therapeutic strategies have been proposed to enhance PARPi sensitivity by inducing HR deficiency or exploiting vulnerabilities in DNA repair, cell-cycle regulation, growth factor signaling, and cellular metabolism (Figure 2). In particular, targeting NAD+ metabolism represents an attractive and underexplored opportunity, given the central role of PARP enzymes in NAD+ consumption and the growing evidence linking metabolic adaptation to PARPi resistance. It is important to highlight that despite the strong mechanistic rationale, many of the sensitization strategies proposed here have not yet undergone CRC-specific clinical evaluation. Most evidence remains preclinical or has been generated in other tumor types, emphasizing that these approaches should currently be viewed as promising investigational strategies rather than clinically established therapies.

Based on the current evidence, we propose a hypothesis-generating framework for future evaluation of PARPis in CRC as a stepwise biomarker-guided strategy (Figure 3). First, patients should be stratified using integrated molecular biomarkers encompassing *TP53*, RAD51, *MRE11*, microsatellite instability, and homologous recombination deficiency signatures. Second, PARPis should be evaluated in rational combination with therapies that either induce a BRCAness phenotype and/or exploit complementary vulnerabilities in DNA repair and cellular metabolism. Among these strategies, targeting metabolic adaptation, particularly NAD+ metabolism, represents a promising and underexplored avenue that complements established DNA damage response-targeted approaches. Finally, prospective biomarker-driven clinical trials should prioritize these combinations in molecularly selected patient populations to determine whether improved PARPi sensitivity translates into meaningful clinical benefit. Such efforts may ultimately expand the therapeutic benefit of PARP inhibition in CRC and facilitate the development of novel biomarker-guided precision oncology approaches.

## Figures and Tables

**Table 1:** Comparison of Selected PARP Inhibitors: Mechanisms, Regulatory Status, and Clinical Uses.

Drug	Selectivity	Mechanism of Action	Regulatory Status and Approved Uses	Adverse Drug Reactions
Olaparib	PARP1, PARP2 [14].	Inhibits PARP catalytic activity by competing with NAD+ at the PARP active site. Promotes PARP trapping on DNA, resulting in accumulation of unrepaired single-strand breaks, replication fork collapse, and synthetic lethality in tumors with homologous recombination repair defects, particularly BRCA1/2-deficient cancers [14,15].	FDA-approved for selected patients with ovarian, breast, pancreatic, and prostate cancers, with indications defined by tumor type, treatment setting, and *BRCA*/HRR/HRD status; certain prostate and ovarian indications involve combination therapy [16,17].	Fatigue, nausea, anemia, vomiting, neutropenia, and thrombocytopenia. Rare but serious adverse events include myelodysplastic syndrome (MDS), acute myeloid leukemia (AML), and pneumonitis [18].
Rucaparib	PARP1, PARP2	Inhibits PARP enzymatic activity and promotes PARP-DNA trapping, preventing repair of DNA damage and inducing synthetic lethality in homologous recombination-deficient tumors [15].	FDA-approved for pre-treated *BRCA*-mutated ovarian cancer treatment and maintenance therapy, as well as *BRCA*-mutated metastatic castration-resistant prostate cancer in patients previously treated with an androgen receptor-directed therapy [15,19].	Fatigue, nausea, anemia, thrombocytopenia, dysgeusia, elevated liver enzymes, and rare cases of MDS/AML [19].
Niraparib	PARP1, PARP2 [20].	Inhibits PARP activity and induces PARP trapping, resulting in replication fork destabilization and accumulation of DNA damage [15,20].	FDA-approved for maintenance treatment of selected adult patients with advanced or recurrent epithelial ovarian, fallopian tube, or primary peritoneal cancer following response to platinum-based chemotherapy, with current indications dependent on treatment setting and *BRCA* status. Niraparib is also available in combination with abiraterone acetate for selected patients with *BRCA*-mutated mCRPC [21,22,23].	Thrombocytopenia, anemia, neutropenia, fatigue, nausea, vomiting, constipation, and insomnia; hematological toxicity requires monitoring and dose adjustment [20,24].
Talazoparib	PARP1, PARP2 [15].	A highly potent PARP inhibitor with strong PARP-trapping activity. Prevents DNA repair and enhances replication-associated DNA damage, producing cytotoxic effects particularly in BRCA1/2-deficient tumors [25].	FDA-approved as single-agent for adult patients with deleterious or suspected deleterious germline *BRCA*-mutated, HER2-negative, locally advanced or metastatic breast cancer. In combination with enzalutamide, talazoparib is also indicated for adult patients with HRR gene-mutated metastatic castration-resistant prostate cancer [26,27,28].	Common toxicities include anemia, neutropenia, thrombocytopenia, fatigue, and nausea. Rare risks include MDS and AML [15].
Veliparib	PARP1, PARP2 [29].	Primarily inhibits PARP catalytic activity with weaker PARP-trapping ability compared with other PARP inhibitors. It has been investigated mainly in combination with chemotherapy due to limited activity as monotherapy [15].	Not currently FDA-approved. Evaluated clinically, predominantly in combination with chemotherapy, across multiple solid tumors [15].	Common adverse effects include nausea, vomiting, fatigue, neutropenia, anemia, and thrombocytopenia, particularly when combined with chemotherapy [15,18].
Fuzuloparib	PARP1, PARP2 [30,31].	Inhibits PARP catalytic activity and promotes PARP trapping, resulting in accumulation of DNA damage and increased cytotoxicity in tumors with impaired DNA repair [30].	NMPA-approved in China for multiple ovarian cancer indications, including treatment of platinum-sensitive recurrent germline *BRCA*-mutated ovarian, fallopian tube, or primary peritoneal cancer after ≥2 prior chemotherapy regimens, with subsequent approvals extending to maintenance settings [30,31].	Common adverse effects include anemia, nausea, fatigue, vomiting, thrombocytopenia, and neutropenia [30].
Pamiparib	PARP1, PARP2 [32].	Inhibits PARP catalytic activity and PARP trapping, leading to accumulation of DNA damage and impaired repair, with enhanced activity in tumors with homologous recombination repair defects [32].	Conditionally approved by the NMPA in China for germline *BRCA*-mutated recurrent advanced ovarian, fallopian tube, or primary peritoneal cancer following ≥2 prior lines of chemotherapy [33].	Common adverse effects include anemia, thrombocytopenia, neutropenia, nausea, fatigue, and vomiting [33,34].

Abbreviations: AML, acute myeloid leukemia; *BRCA*, breast cancer susceptibility gene; FDA, U.S. Food and Drug Administration; HER2, human epidermal growth factor receptor 2; HRD, homologous recombination deficiency; HRR, homologous recombination repair; mCRPC, metastatic castration-resistant prostate cancer; MDS, myelodysplastic syndrome; NAD+, nicotinamide adenine dinucleotide; NMPA, National Medical Products Administration (China); PARP, poly(ADP-ribose) polymerase.

**Table 2:** Summary of clinical trials on CRC-specific cohorts.

Trial Identifier/Code	Phase	Setting	Biomarker Selection	PARPi Regimen	Sample Size	Primary Endpoint	Efficacy Result	Key Toxicities	Study Status	Reference
NCT00535353	I	Previously treated locally advanced/mCRC	None	Olaparib + irinotecan	26 enrolled, 25 treated	Safety, DLT, recommended dose/schedule	No ORR; SD in 9 patients. Overall antitumor activity limited.	Fatigue, anorexia, diarrhea, nausea/vomiting, neutropenia, thrombocytopenia; continuous olaparib dosing was poorly tolerated necessitating intermittent dosing.	Completed	[89]
NCT00912743	II	Previously Treated Patients With Stage IV, Measurable CRC	Patients stratified by MSI status, but not selected for HRD	Olaparib monotherapy	33	Tumor response/time to progression	No CR/PR; median PFS ≈ 1.84 months; no meaningful benefit in either MSS or MSI-H CRC.	Mainly nausea, fatigue, vomiting; generally manageable.	Completed	[90]
NCT01051596	II	Heavily pretreated mCRC	None required; MGMT/PTEN explored	Veliparib + temozolomide	75	Disease-control rate	DCR ≈ 24%; 2 confirmed PRs (2.7%); median PFS ≈ 1.8 months and OS ≈ 6.6 months.	Predominantly hematologic; thrombocytopenia was the principal grade ≥ 3 toxicity.	Completed	[91]
NCT01589419	Ib	Neoadjuvant locally advanced rectal cancer	None	Veliparib + capecitabine + pelvic radiation therapy	32 enrolled, 31 efficacy-evaluable	Safety, DLT, RP2D	Tumor downstaging 71%; pCR 29%	Nausea, diarrhea and fatigue common; DLTs included radiation skin injury and nausea/vomiting.	Completed	[92]
NCT02305758	II randomized	Previously untreated mCRC	None	Veliparib + FOLFIRI ± bevacizumab vs. placebo + FOLFIRI ± bevacizumab	130	PFS	No benefit: median PFS 12 vs. 11 months (HR 0.94, 95% CI 0.60–1.48); median OS 25 vs. 27 months (HR 1.26, 95% CI 0.74–2.16); ORR 57% vs. 62%.	More cytopenias with veliparib; grade 3/4 neutropenia 59% vs. 22%; hematopoietic cytopenias 79% vs. 52%.	Completed	[88]
NCT02921256	II randomized platform	High-risk stage II/III locally advanced rectal cancer	No PARPi biomarker selection	Total neoadjuvant therapy (TNT) + Veliparib vs. TNT	178 in veliparib comparison (90 veliparib, 88 control)	Neoadjuvant Rectal (NAR) score	No significant benefit. Mean NAR ≈ 13.3 vs. 12.5 long-term; 3-y DFS ≈ 60% vs. 67%; 3-y OS ≈ 85% vs. 92%. pCR numerically higher but not significant.	No unexpected safety signal	Veliparib arm completed	[93]
NCT03851614 DAPPER	II randomized basket trial	Previously treated pMMR/MSS mCRC, CRC cohort within randomized basket trial	pMMR/MSS	Durvalumab + olaparib vs. durvalumab + cediranib	31 metastatic pMMR-CRC: 16 to durvalumab + olaparib and 15 to durvalumab + cediranib	Immune/TME changes and safety/activity	No ORR in CRC; only isolated SD; PARPi-containing arm did not demonstrate meaningful clinical activity.	Generally manageable; anemia among relevant grade ≥ 3 events; no unexpected PARPi safety signal.	CRC cohort completed	[94]
NCT03983993 NIPAVect	II	Previously treated advanced/mCRC	RAS wild-type	Niraparib + panitumumab	26 enrolled; 24 evaluable	Clinical benefit/activity	ORR 25% (6 PR); 14 additional SD; CBR 83.3%; median PFS ≈ 5.6 months; median OS ≈ 20.9 months.	Rash 58.4%, mucositis, dry skin; grade 3 anemia 12.5%; grade 3 dermatitis, mucositis, HTN and neutropenia ~4.2% each; no grade 4/5 TRAE.	Active, Not recruiting	[95]
NCT04166435	II	Advanced/mCRC	MGMT promoter hypermethylation; low MGMT protein subsequently identified as exploratory marker	Temozolomide + olaparib	11 enrolled; 9 efficacy-evaluable	ORR	ORR 0%, 5/9 patients achieved SD (DCR 55.6%).	Combination considered generally tolerable; no major unexpected safety signal reported.	Completed; study stopped after the predefined efficacy stopping rule was met.	[96]
NCT04456699 LYNK-003	III randomized	Maintenance after first-line FOLFOX + bevacizumab in unresectable/mCRC without progression	None	Olaparib + bevacizumab vs. olaparib vs. bevacizumab + fluoropyrimidine	309	PFS	Median PFS 3.7, 3.5, and 5.6 months, respectively; HR 1.52 (olaparib + bevacizumab vs. control) and 2.11 (olaparib vs. control); no clinical benefit from either olaparib-containing regimen.	Treatment-related AEs: 62% (olaparib + bevacizumab), 50% (olaparib), and 59% (control); no new safety signals or treatment-related deaths.	Stopped early for futility at prespecified interim PFS analysis	[87]
NCT04511039	I	Second-line or later (≥2L) locally advanced/metastatic CRC or gastroesophageal adenocarcinoma	None required for dose escalation; CRC dose-expansion cohorts selected for *TP53*-mutant tumors and stratified by RAS-mutant or RAS wild-type status	Trifluridine/tipiracil (TAS-102) + talazoparib	45 planned	Safety, DLT, MTD/RP2D	No mature efficacy result available as of August 2026.	Not reported.	Recruiting as of 2026 registry reporting	[97]
NCT04926324	Ib/II	Preoperative locally advanced resectable rectal cancer	None required	Niraparib + dostarlimab + hypofractionated radiation therapy	3 actually enrolled	Phase Ib: safety/MTD; phase II: efficacy	Too few patients for efficacy assessment.	Diarrhea, colitis, cytopenias occurred during phase Ib.	Terminated during phase Ib because of treatment-related toxicity	[98]
NCT05201612	II	Refractory advanced/mCRC	HRD: deleterious BRCA alteration and/or low RAD51 score (<10%) per protocol	Pembrolizumab + olaparib	40 planned	ORR by RECIST 1.1	No mature efficacy result available as of August 2026.	Not reported	Unknown, last known status was “Recruiting” in 02-2023	[99]
NCT05732129	II	Second-line HRD-positive mCRC after first-line failure	HRD-positive	Fluzoparib + irinotecan	29 planned	ORR; PFS/OS secondary	No mature efficacy result available as of August 2026.	Not reported	Unknown, last known status was “Not yet recruiting” in 02-2023	[100]
NCT06365970	II	Advanced dMMR/MSI-H CRC after progression on PD-1–based immunotherapy	dMMR/MSI-H	Niraparib + dostarlimab	20 planned; 0 participants enrolled	ORR	Not evaluable	None evaluable	Withdrawn	[101]
NCT00516438	I	Advanced solid tumors, including CRC	None	Olaparib + topotecan	21 enrolled; 19 treated overall; CRC n = 8	Safety, DLT, MTD	No responses among 8 CRC patients; combination development was not pursued because tolerable doses resulted in subtherapeutic exposure	Dose-limiting neutropenia and thrombocytopenia; fatigue and gastrointestinal AEs	Completed	[102]

Abbreviations: CBR, clinical benefit rate; CR, complete response; CRC, colorectal cancer; DCR, disease control rate; DFS, disease-free survival; DLT, dose-limiting toxicity; dMMR, deficient mismatch repair; FOLFIRI, folinic acid, fluorouracil, and irinotecan; FOLFOX, folinic acid, fluorouracil, and oxaliplatin; HRD, homologous recombination deficiency; MGMT, O^6^-methylguanine-DNA methyltransferase; mCRC, metastatic colorectal cancer; MSI-H, microsatellite instability-high; MSS, microsatellite-stable; MTD, maximum tolerated dose; NAR, neoadjuvant rectal; ORR, objective response rate; OS, overall survival; PARPi, PARP inhibitor; pCR, pathological complete response; PD-1, programmed cell death protein 1; PFS, progression-free survival; pMMR, proficient mismatch repair; PR, partial response; RAS, rat sarcoma virus; RECIST, Response Evaluation Criteria in Solid Tumors; RP2D, recommended phase II dose; SD, stable disease; TME, tumor microenvironment; TNT, total neoadjuvant therapy; TRAE, treatment-related adverse event.

## Data Availability

No new data were created or analyzed in this study. Data sharing is not applicable to this article.
